# Supplementary material for: Deep learning for fully automatic detection, segmentation, and Gleason grade estimation of prostate cancer in multiparametric magnetic resonance images
Source: Sci Rep. 2022 Feb 22;12:2975. doi: 10.1038/s41598-022-06730-6 (PMC8864013; doi:10.1038/s41598-022-06730-6)
Supplement: Supplementary file 1 — Supplementary Information. [file 41598_2022_6730_MOESM1_ESM.pdf]

Supplementary material: Deep Learning for fully automatic detection, segmentation, and Gleason Grade estimation of prostate cancer in multiparametric Magnetic Resonance Images

Oscar J. Pellicer-Valero*[1], José L. Marenco Jiménez[2], Victor Gonzalez-Perez[3], Juan Luis Casanova Ramón-Borja[2], Isabel Martín García[4], María Barrios Benito[4], Paula Pelechano Gómez[4], José Rubio-Briones[2], María José Rupérez[5], and José D. Martín-Guerrero[1]

[1]Intelligent Data Analysis Laboratory, Department of Electronic Engineering, ETSE (Engineering School), Universitat de València (UV), Av. Universitat, sn, 46100 Bujassot, Valencia, Spain. E-Mails: `Oscar.Pellicer@uv.es (+34 9635 44022)`, `jose.d.martin@uv.es`

[2]Department of Urology, Fundación Instituto Valenciano de Oncología (FIVO), Beltrán Báguena, 8, 46009 Valencia, Spain. E-Mails: `jlmarencoj@gmail.com`, `jcasanova@fivo.org`, `jrubio@fivo.org`

[3]Department of Medical Physics, Fundación Instituto Valenciano de Oncología (FIVO), Beltrán Báguena, 8, 46009 Valencia, Spain. E-Mails: `vgonzalezper@hotmail.com`

[4]Department of Radiodiagnosis, Fundación Instituto Valenciano de Oncología (FIVO), Beltrán Báguena, 8, 46009 Valencia, Spain. E-Mails: `mismaga99@gmail.com`, `mar7esc@gmail.com`, `ppelechano@hotmail.com`

[5]Instituto de Ingeniería Mecánica y Biomecánica, Universitat Politècnica de València (UPV), Camino de Vera, sn, 46022, Valencia, Spain. E-Mail: `mjrupere@upvnet.upv.es`

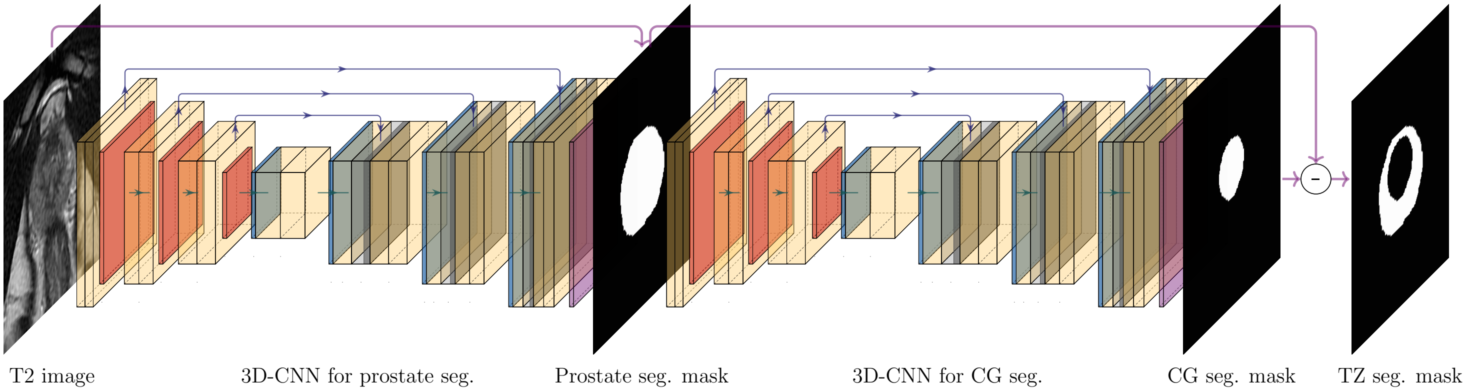

Figure 1: Cascading three-dimensional (3D) convolutional neural networks (CNNs) for prostate central gland (CG) and peripheral zone (PZ) segmentation. The first 3D-CNN takes a T2 sequence as input and produces a prostate segmentation mask as output, while the second 3D-CNN takes both the T2 sequence and the prostate segmentation (generated by the previous CNN) as inputs to produce the CG segmentation mask as output. Finally, PZ is computed by subtraction of both output masks.

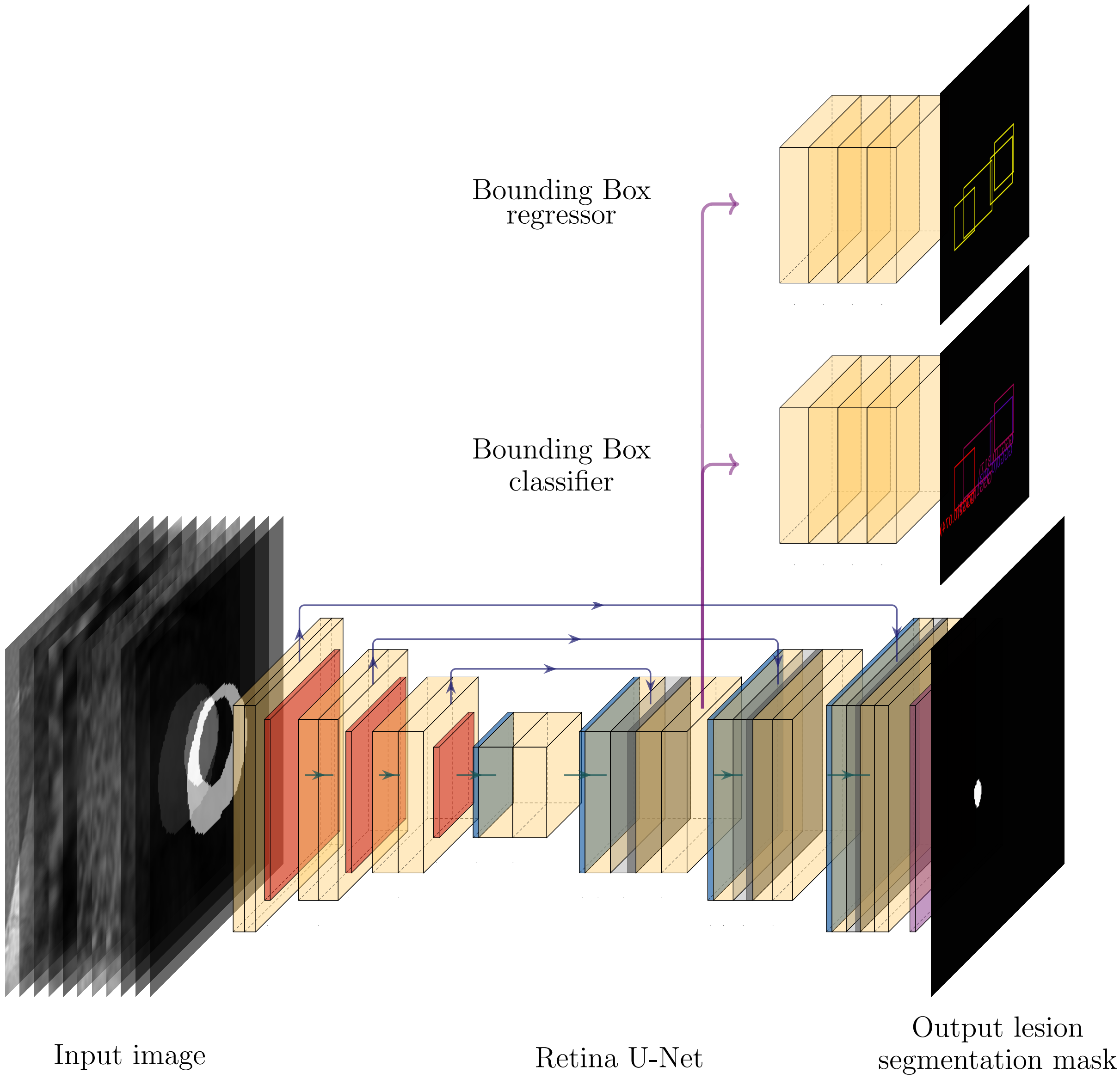

Figure 2: Overview of the Retina U-Net architecture. On the bottom, a U-Net-like architecture segments the lesions present in the image irrespective of their class. On the top, a bounding box (BB) regression head takes a feature map from a decoder of the U-Net and refines the coarse detections, while the BB classifier tries to predict their class. These two heads visit all decoder levels, performing detection at different scales transparently.

Table 1: Quantitative results for IVO (top) and ProstateX (bottom) test data evaluated with different Gleason Grade Group (GGG) significance criteria (e.g.: lesions with GGG≥1, 2, or 3 are considered positive), at lesion- and patient-level ($N_{positives}/N_{total}$), and at two thresholds (t): maximum sensitivity and balanced (same thresholds as Table 1 in the main text). Results here are expressed in terms of positive predictive value (PPV) and negative predictive value (NPV). For IVO data, results are compared with radiologist-assigned pre-biopsy PI-RADS scores for all IVO patients with no missing sequences and with PI-RADS information available (N=106 patients, 111 lesions). AUC: Area under the ROC curve.

(Dataset) & Significance criterion	Level	AUC	Max. sensitivity			Balanced			PI-RADS≥4		PI-RADS=5	
			t	PPV	NPV	t	PPV	NPV	PPV	NPV	PPV	NPV
(IVO) GGG≥1	Lesion (13/33)	0.892	0.027	0.500	1.000	0.105	0.667	0.933	0.672	0.681	0.905	0.567
	Patient (15/30)	0.920	0.253	0.750	1.000	0.301	0.812	0.857	0.790	0.545	0.952	0.424
**GGG≥2**	Lesion (8/33)	0.945	0.173	0.615	1.000	0.301	0.700	0.957	0.469	0.915	0.714	0.789
	Patient (9/30)	0.910	0.219	0.643	1.000	0.262	0.667	0.944	0.548	0.864	0.762	0.718
GGG≥3	Lesion (3/33)	0.856	0.301	0.333	1.000	0.315	0.333	0.963	0.125	0.936	0.238	0.933
	Patient (3/30)	0.840	0.301	0.333	1.000	0.315	0.333	0.958	0.129	0.932	0.238	0.929
(ProstateX) GGG≥1	Lesion (17/69)	0.898	0.028	0.593	0.976	0.053	0.667	0.938	-	-	-	-
	Patient (16/45)	0.866	0.028	0.390	1.000	0.104	0.600	0.950	-	-	-	-
**GGG≥2**	Lesion (13/69)	0.959	0.028	0.520	1.000	0.108	0.706	0.981	-	-	-	-
	Patient (13/45)	0.865	0.028	0.394	1.000	0.108	0.545	0.957	-	-	-	-
GGG≥3	Lesion (7/69)	0.751	0.195	0.417	0.965	0.195	0.417	0.965	-	-	-	-
	Patient (7/45)	0.767	0.016	0.233	1.000	0.026	0.240	0.950	-	-	-	-

Table 2: Quantitative results for IVO (top) and ProstateX (bottom) test data evaluated with different Gleason Grade Group (GGG) significance criteria (e.g.: lesions with GGG≥1, 2, or 3 are considered positive), at lesion- and patient-level ($N_{positives}/N_{total}$), and at two thresholds (t): maximum test sensitivity (which is the lesion-level maximum sensitivity threshold for GGG≥2 classification in the test set, but applied to all GGGs), and the maximum train sensitivity (same as the previous one, but the threshold was obtained from the training data). Results are expressed in terms of Area under the ROC curve (AUC), sensitivity, specificity, positive predictive value (PPV) and negative predictive value (NPV).

(Dataset) & Significance criterion	Level	AUC	Max. train sensitivity					Max. test sensitivity				
			t	Sens.	Spec.	PPV	NPV	t	Sens.	Spec.	PPV	NPV
(IVO) GGG≥1	Lesion (13/33)	0.892	0.164	0.846	0.800	0.733	0.889	0.173	0.846	0.800	0.733	0.889
	Patient (15/30)	0.920	0.164	1.000	0.067	0.517	1.000	0.173	1.000	0.200	0.556	1.000
**GGG≥2**	Lesion (8/33)	0.945	0.164	1.000	0.600	0.444	1.000	0.173	1.000	0.800	0.615	1.000
	Patient (9/30)	0.910	0.164	1.000	0.571	0.500	1.000	0.173	1.000	0.571	0.500	1.000
GGG≥3	Lesion (3/33)	0.856	0.164	0.714	0.855	0.357	0.964	0.173	1.000	0.667	0.231	1.000
	Patient (3/30)	0.840	0.164	0.714	0.711	0.312	0.931	0.173	1.000	0.630	0.231	1.000
(ProstateX) GGG≥1	Lesion (17/69)	0.898	0.086	0.706	0.923	0.750	0.906	0.028	0.941	0.788	0.593	0.976
	Patient (16/45)	0.866	0.086	0.938	0.586	0.556	0.944	0.028	1.000	0.138	0.390	1.000
**GGG≥2**	Lesion (13/69)	0.959	0.086	0.923	0.893	0.667	0.980	0.028	1.000	0.786	0.520	1.000
	Patient (13/45)	0.865	0.086	0.923	0.625	0.500	0.952	0.028	1.000	0.375	0.394	1.000
GGG≥3	Lesion (7/69)	0.751	0.086	1.000	0.667	0.231	1.000	0.028	0.714	0.742	0.238	0.958
	Patient (7/45)	0.767	0.086	1.000	0.630	0.231	1.000	0.028	0.714	0.500	0.208	0.905
